# Neighborhood Socioeconomic Status and the Functional Outcome of Patients Treated With Endovascular Thrombectomy for Ischemic Stroke

**DOI:** 10.1212/WNL.0000000000213615

**Published:** 2025-06-13

**Authors:** Bridget A. Schoon, Daniël Hansen, Bob Roozenbeek, Joost Oude Groeniger, Wouter van der Steen, Aad van der Lugt, Manon Kappelhof, Yvo B.W.E.M. Roos, Charles B.L.M. Majoie, Frank van Lenthe, Diederik W.J. Dippel, for the MR CLEAN-MED and MR CLEAN-NO IV investigators

**Affiliations:** 1 Department of Neurology, Erasmus MC University Medical Center, Rotterdam, the Netherlands;; 2 Department of Radiology and Nuclear Medicine, Erasmus MC University Medical Center, Rotterdam, the Netherlands;; 3 Department of Public Health, Erasmus MC University Medical Center, Rotterdam, the Netherlands;; 4 Department of Radiology and Nuclear Medicine, Amsterdam University Medical Center, University of Amsterdam, the Netherlands;; 5 Department of Radiology and Nuclear Medicine, OLVG Hospital, Amsterdam, the Netherlands; and; 6 Department of Neurology, Amsterdam University Medical Center, University of Amsterdam, the Netherlands.

## Abstract

**Background and Objectives:**

Socioeconomically deprived neighborhoods are known to have higher incidence rates of stroke and less access to high-quality stroke care. We aimed to examine whether there is an association between neighborhood socioeconomic status (nSES) and functional outcome after endovascular thrombectomy (EVT) for ischemic stroke in a high-income country.

**Methods:**

Data from 2 randomized trials, which included patients treated with EVT within 6 hours after stroke onset: MR CLEAN-MED and MR CLEAN-NO IV were studied. A per postcode composite score of education, employment, and household income (scores ranging from −1 to 1) created by Statistics Netherlands, represented nSES. The association with functional outcome after 90 days (modified Rankin Scale [mRS]), functional independence (mRS 0–2), neurologic deficit at 24 hours (NIH Stroke Scale [NIHSS]), and radiologic outcomes (expanded treatment in cerebral infarction score and follow-up infarct volume [FIV]) were analyzed using regression analyses adjusted for patient characteristics, including baseline NIHSS.

**Results:**

We included 910 patients (median age 71.5 years, 404 (44.4%) women, median baseline NIHSS 15) in the analyses. Patients with a higher nSES had a higher likelihood of a more favorable functional outcome (a shift toward improved outcome on the mRS) (adjusted common odds ratio [OR] 1.90, 95% CI 1.21–3.01) and were more likely to have regained functional independence (adjusted OR 3.21, 95% CI 1.82–5.70) at 90 days. There was no significant association between the nSES and the degree of neurologic deficit at 24 hours (adjusted β −0.24, 95% CI −0.50 to 0.01, *p* = 0.06) or radiologic outcomes (reperfusion status [adjusted OR 0.89, 95% CI 0.45–1.78], FIV [adjusted β 0.01, 95% CI −0.17 to 0.20, *p* = 0.89]).

**Discussion:**

Living in a more socioeconomically affluent neighborhood was associated with a more favorable functional outcome at 90 days, but not with degree of neurologic deficit at 24 hours or radiologic outcomes. This suggests that nSES-based inequalities exist in the postacute phase of stroke care, and highlights the importance of continuing to work toward health equity for patients with stroke.

## Introduction

Patients with stroke living in more socioeconomically disadvantaged neighborhoods continue to have less access to optimal stroke care,^1-3^ less favorable functional outcomes,^4^ and higher poststroke mortality than their more affluent counterparts.^5,6^ However, few studies have examined the association between socioeconomic factors and functional outcome after endovascular thrombectomy (EVT) in patients who were functionally independent before their index stroke.^7,8^

In the Netherlands, patients with a stroke caused by a large vessel occlusion eligible for EVT are treated in one of the country's EVT-capable or comprehensive stroke centers. Stroke care in the Netherlands is monitored through the Dutch Acute Stroke Audit, which reviews the quality of care provided on both the patient and hospital levels.^9^ These patients receive comparable care as stipulated in national and international protocols, which are endorsed by the Dutch societies for neurology and radiology. Therefore, we do not expect that there will be a significant association between neighborhood socioeconomic status (nSES) and the in-hospital outcome parameters examined. After discharge, however, the rehabilitation care and follow-up treatment received may be much more variable and could be influenced by the patients' socioeconomic environment. In this study, we aimed to examine whether there is an association between nSES and functional outcome after EVT among patients with ischemic stroke living in regions with varying nSES within a high-income country.

## Methods

### Study Design and Population

In this post hoc study, we analyzed data collected in 2 previously published randomized clinical trials (RCTs): MR CLEAN-MED and MR CLEAN-NO IV.^10,11^ These RCTs were phase III multicenter clinical trials, with randomized group assignment, open label treatment, and blinded outcome evaluation. MR CLEAN-MED compared treatment with EVT with periprocedural use of intravenous antithrombotics (aspirin and/or unfractionated heparin) vs standard-of-care treatment with EVT. MR CLEAN-NO IV compared treatment with intravenous thrombolytics followed by EVT vs EVT alone.

The trials enrolled patients who presented at one of the participating EVT-capable or comprehensive stroke centers in the Netherlands between 2018 and 2021. MR CLEAN-NO IV also enrolled patients in French and Belgian centers during this period. Both trials had a similar design and follow-up procedure, including assessment of National Institutes of Health Stroke Scale (NIHSS) score at baseline and 24-hour postrandomization, and assessment of functional outcome, as represented by the modified Rankin Scale (mRS) approximately 3 months (90 ± 14 days) postrandomization.

Patients who met the inclusion criteria for the RCTs were aged 18 or older, functionally independent before their index stroke (prestroke mRS 0–2), had a stroke caused by an EVT-eligible anterior circulation large vessel occlusion, and presented to the emergency department within 4.5 hours (MR CLEAN-NO IV) or 6 hours (MR CLEAN-MED) since stroke onset or last-seen-well time. A more detailed description of the inclusion and exclusion criteria of the RCTs is available in the study protocols.^12,13^

Since nSES was required to perform the analyses, patients could only be included in this cohort if they were residing in the Netherlands and had a Dutch postcode at the time of their index stroke. Postcodes were routinely collected during the patients' participation in the trials.

### Standard Protocol Approvals, Registrations, and Patient Consents

Ethical approval for these post hoc analyses was obtained from the medical ethical review board of the Erasmus MC, University Medical Center in Rotterdam, the Netherlands (MEC-2022-0787). All patients included in this study indicated that they did not object to the use of their pseudonymized data for further medical research when signing informed consent for participation in the aforementioned clinical trials (MR CLEAN-NO IV, MR CLEAN MED).

### Covariates

#### Patient Characteristics

Data collected on the patients' demographic characteristics, including age, sex, comorbidities (as listed in Table 1), and stroke severity at baseline (as represented by the NIHSS score) were sourced from the MR CLEAN-MED and MR CLEAN-NO IV data sets.

**Table 1:** Baseline and Procedural Characteristics of the Total Cohort

Patient characteristics	Total cohort (n = 910)	Missing, n (%)
Age, y, median (IQR)	71.5 (62 to 79)	0
Sex, male, n (%)	506 (55.6)	0
Prestroke mRS, n (%)		
mRS 0	647 (71.1)	0
mRS 1	175 (19.2)	
mRS 2	88 (9.7)	
nSES, SES-WOA score, median (IQR)	0.0 (−0.2 to 0.2)	4 (0.4)
Medical history, n (%)		
Stroke	153 (16.8)	0
Hypertension	419 (46.0)	0
Hypercholesterolemia	209 (23.0)	0
Diabetes mellitus	141 (15.5)	0
Smoking	218 (25.5)	55 (6.0)
Atrial fibrillation	158 (17.4)	0
Congestive heart failure	58 (6.4)	0
Peripheral artery disease	57 (6.3)	0
Myocardial infarction	100 (11.0)	0
BMI, kg/m^2^, median (IQR)	25.4 (23.3 to 28.3)	124 (13.6)
Onset to groin time, min, median (IQR)	155 (120 to 205)	46 (5.1)
Door to groin time, min, median (IQR)	48 (30 to 66)	47 (5.2)
Baseline NIHSS, median (IQR)	15 (9 to 19)	0
ASPECTS, median (IQR)	9 (8 to 10)	2 (0.2)
Occlusion location, n (%)		
ICA	217 (23.8)	3 (0.3)
M1	495 (54.3)	
M2	187 (20.5)	
None	8 (0.8)	
Anesthesia management, n (%)		
Local only	608 (66.8)	43 (4.7)
Local and bolus opiates	78 (8.6)	
Moderate sedation	68 (7.5)	
Deep sedation	26 (3.1)	
General anesthesia	87 (9.6)	

Abbreviations: ASPECTS = Alberta Stroke Program Early CT Score; BMI = body mass index; ICA = internal carotid artery; IQR = interquartile range; mRS = modified Rankin Scale; NIHSS = NIH Stroke Scale; nSES = neighborhood socioeconomic status.

#### Neighborhood Socioeconomic Status

The SES-WOA score is a composite measure used to represent nSES, based on 3 key components: prosperity (welvaart), educational level (opleidingsniveau), and employment history (arbeidsverleden), collectively abbreviated as WOA. The SES-WOA is a publicly available composite score of the level of education achieved, household income, and employment status of an inhabited area in the Netherlands that is presented per 4-digit postcode.^14^ It was created using multiple correspondence analysis of Dutch census data. The SES-WOA score ranges between −1 and 1, where a lower score indicates a more socioeconomically deprived area. A more detailed description of the development of the composite score is available online.^15^

### Outcomes

#### Degree of Neurologic Deficit

The degree of neurologic deficit at 24 hours is represented by the NIHSS score, which was obtained 24-hour postrandomization in the MR CLEAN-MED or MR CLEAN-NO IV trials.

#### Radiologic Outcomes

The reperfusion status postthrombectomy was scored with the expanded treatment in cerebral infarction (eTICI) score derived from digital subtraction angiography imaging acquired during the procedure. Patients with an eTICI of 2b–3 were considered to have successful reperfusion, and a score of 0–2a was considered unsuccessful.

The follow-up infarct volume (FIV) in milliliters was calculated from either an MRI at 24-hour postrandomization or noncontrast CT at 5–7-day postrandomization.

#### Functional Outcome

The functional outcome as represented by the mRS (ranging from 0, no remaining symptoms, to 6, death) was collected at 90 days (±14 days) as part of the final patient assessment of the MR CLEAN-MED and MR CLEAN-NO IV trials through standardized interviews performed by trained research nurses. Functional independence was defined as mRS 0–2. As part of the trial protocols, a committee masked for treatment allocation adjudicated the functional outcome based on the information described in interview reports.

### Statistical Analysis

All analyses were completed in R (version 4.2.1: “Funny-Looking Kid”). Baseline data of the patients from the merged data sets included in the study will be reported according to standard statistical procedures. Conventional levels of significance (α 0.05) were used for interpretation. Adjusted and unadjusted estimates of the regression analyses are reported with corresponding 95% CIs.

#### Association Between nSES and the Degree of Neurologic Deficit

The association between nSES and NIHSS score at 24 hours was examined using linear regression. The NIHSS was log-transformed to account for potential issues with the skewed distribution of the data. The analysis was adjusted for baseline NIHSS, age, and sex. Additional adjustment for the periprocedural anesthesia method was performed to account for the potential effect of (general) anesthesia on the patients' neurologic status at 24 hours. To examine the potential influence of clustering of patients within hospitals, a sensitivity analysis was performed using linear regression with generalized estimating equations (GEE) methods.

#### Association Between nSES and Radiologic Outcomes

The association between nSES and postprocedural reperfusion status (dichotomized eTICI score) was examined through binary logistic regression. The association between the nSES and the FIV was examined using linear regression. The FIV was log-transformed to account for potential issues with the skewed distribution of the variable. Both analyses were adjusted for sex, age, and baseline occlusion location.

#### Association Between nSES and Functional Outcome

The association between nSES (SES-WOA score) and the mRS at 90 days is represented by the common odds ratio (cOR) and 95% CI, estimated with ordinal logistic regression. This represents the common odds for a 1-step shift in the mRS score toward improved outcome. The analysis was performed with adjustment for baseline NIHSS, sex, and age. Additional adjustment for comorbidities to the analysis was examined separately. Adherence of the ordinal regression model to the proportional odds assumption was examined.

The association between nSES and functional independence is represented by the odds ratio (OR), estimated with binomial logistic regression. The analysis was performed adjusting for the baseline NIHSS, sex, and age. To examine the potential influence of clustering of patients within hospitals, a sensitivity analysis was performed with logistic regression estimated using GEE methods.

To further visualize the differences in functional outcome between patients with varying nSES, the study participants were divided into quartiles of near equal size based on their SES-WOA score. The 4 categories were labelled as low nSES, middle-low nSES, middle-high nSES, and high nSES. The absolute percentage difference of the number of patients who regained functional independence (mRS 0–2) between categories is reported. The baseline characteristics of each nSES quartile are reported separately.

#### Missing Data

Missing data were imputed through multivariate imputation by chained equations (5 sets) using the “MICE” package in the statistical software R (version 4.2.1).

### Data Availability

Pseudonymized clinical data used for the analyses in the article will be made available on reasonable request. Data access requests should be submitted through the CONTRAST consortium website.

## Results

**Figure 1:**
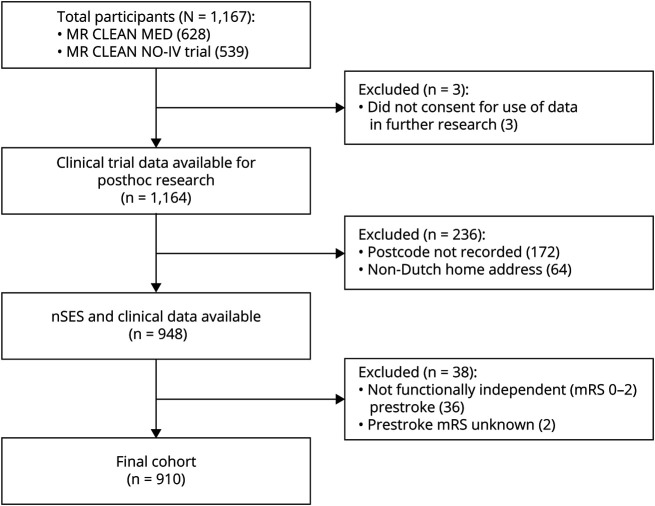
Study Profile mRS = modified Rankin Scale; nSES = neighborhood socioeconomic status.

Of the 1,167 patients originally included in MR CLEAN-MED and MR CLEAN-NO IV, 910 met the criteria for inclusion in this study's final analyses (Figure 1). Two hundred thirty-six patients were excluded because of unavailability of postcode data of whom 64 were excluded because of having a home address outside of the Netherlands. A further 38 patients were excluded because they were not functionally independent (mRS 0–2) before their index stroke. An overview of the baseline characteristics and procedural outcomes of the included patients and the patients excluded because of the absence of a recorded postcode are available in eTable 1 in eAppendix 1.

The included patients had a median age of 71.5 (interquartile range [IQR] 62–79), and a little over half (55.6%) were men. The median NIHSS score at baseline was 15 (IQR 9–19). Participants resided in 617 different Dutch postcodes, the 4 most common postcodes occurred 6 times. The median nSES was 0.00, with a maximum score of 0.53 and a minimum score of −0.86. Table 1 summarizes further relevant baseline and procedural (EVT) characteristics of the cohort, including the number of missing values for each variable.

### Association Between nSES and Degree of Neurologic Deficit

There were 24 (2.6%) missing values for the NIHSS at 24 hours. If the patient had died before the 24-hour postprocedure time point, the NIHSS was imputed as 38, a score that a deeply comatose patient, who would not respond to any stimulus, could realistically have.^16^ The remaining missing NIHSS values were imputed with multiple imputation. The patients' baseline characteristics are reported per the anesthesia method used in eTable 2.

The median NIHSS at 24 hours for the cohort was 6 (IQR 1–13). The regression coefficient (β) for the association with nSES for the adjusted analysis was −0.24 (95% CI −0.50 to 0.01, *p* = 0.06) (Table 2). Since the NIHSS was log transformed, this regression coefficient indicates that for a 1-unit increase in the SES-WOA score, the NIHSS changes by a factor of 0.78 (exponent of −0.24) or 22%. This implies that there is a no trend toward a lower NIHSS at 24 hours for participants with a higher nSES. A sensitivity analysis was preformed to explore the influence of clustering of patients within hospitals. The results were very similar (aβ −0.24, 95% CI −0.49 to 0.01, *p* = 0.06) to the main analysis.

**Table 2:** Associations Between Outcome Measures and nSES

	Unadjusted (95% CI)	Adjusted (95% CI)
mRS at 90 d (cOR)	1.80 (1.15 to 2.83)	1.90 (1.21 to 3.01)
mRS 0–2 at 90 d (OR)	2.58 (1.54 to 4.37)	3.21 (1.82 to 5.70)
NIHSS at 24 h (β)^a^	−0.21 (−0.49 to 0.07)	−0.24 (−0.50 to 0.01)
eTICI score (OR)	0.97 (0.52 to 2.05)	0.89 (0.45 to 1.78)
FIV (β)^b^	0.04 (−0.16 to 0.26)	0.01 (−0.17 to 0.20)

Abbreviations: cOR = common OR; eTICI = expanded treatment in cerebral infarction; FIV = follow-up infarct volume; mRS = modified Rankin Scale; NIHSS = NIH Stroke Scale; nSES = neighborhood socioeconomic status; OR = odds ratio.

a Analysis with log-transformed NIHSS, β indicates a nonsignificant change in NIHSS at 24 hours by a factor of 0.78 per 1 point change in the SES-WOA score.

b Analysis with log-transformed FIV, β indicates a nonsignificant change in the FIV by a factor of 1.013 per 1 point change in the SES-WOA score.

### Association Between nSES and Radiologic Outcomes

Successful reperfusion status was achieved in 81.3% of patients. Eighty (8.8%) of the eTICI scores were missing. There was no statistically significant association between nSES and the reperfusion status (adjusted OR [aOR] 0.89, 95% CI 0.45–1.78) (Table 2).

The median FIV was 20 mL (IQR 5–68). One hundred thirty (14.3%) of the values were missing. The adjusted regression coefficient was 0.01 (95% CI −0.17 to 0.20, *p* = 0.89) (Table 2). This represents a nonsignificant 1.3% (factor of 1.013) change in the FIV per 1 point change in the SES-WOA score (which ranges from −1 to 1).

### Association Between nSES and Functional Outcome

The analysis yielded an adjusted cOR of 1.90 (95% CI 1.21–3.01), indicating that the odds of being in a lower (more favorable) mRS category significantly increases by a factor 1.9 with a 1-point increase in nSES (Table 2). We chose not to adjust for comorbidities when performing the main analysis. However, the effect of additional adjustment for comorbidities to the analysis was minor as shown in eTable 3 in eAppendix 2.

The Brant test performed on the adjusted model was statistically significant (*p* = 0.00) indicating that the proportional odds assumption was violated in this analysis. Adjusted and unadjusted ORs for each dichotomization of the mRS are reported to further visualize this concept in eTables 4–6 in eAppendix 3. Although the effect was not proportional at each cut-point, the shift is consistently in the same direction.

As the nSES increased, the likelihood of regaining functional independence increased significantly (aOR 3.21, 95% CI 1.82–5.70). The adjusted and unadjusted ORs for the binomial logistic regression are reported (Table 2). A sensitivity analysis was preformed to explore the influence of clustering of patients within hospitals. The results were very similar (aOR 3.21, 95% CI 1.44–4.18) to the main analysis.

We created 4 near equal groups of patients based on nSES quartiles. Baseline and procedural characteristics of the quartiles are displayed in eTable 7. The sex distribution as well as the median age and baseline NIHSS did not differ significantly between quartiles. However, significantly more patients in the low-nSES quartile had a history of diabetes mellitus (8.8% in the high vs 21% in the low nSES quartile) and had a higher median body mass index (BMI). There were no significant differences in the recorded procedural characteristics.

**Figure 2:**
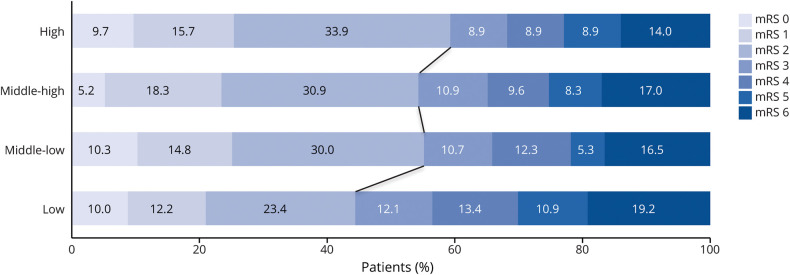
Distribution of the mRS Score at 90 Days per nSES Category mRS = modified Rankin Scale; nSES = neighborhood socioeconomic status.

The distribution of the mRS at 90 days for each nSES is displayed for visualization (Figure 2). In total, 59.3% of the high, 54.4% of the middle-high, 55.1% of the middle low, and 44.3% of the low quartile retained functional independence at 90-day post-EVT. This resulted in an absolute difference between those in the highest quartile and the other quartiles of 4.9%, 0.7%, and 10.8%, respectively.

## Discussion

In this post hoc study with data from 910 patients treated with EVT for acute ischemic stroke from 2 randomized trials, we found an association between the patients' nSES and functional outcome at 90-day posttreatment. We included patients who were functionally independent before their index stroke and found that patients with a lower nSES had a decreased likelihood of regaining functional independence within 90 days than their more affluent counterparts.

There was no statistically significant association between nSES and the degree of neurologic deficit at 24 hours. The result of the linear regression analysis examining this association indicated a nonsignificant 22% change in the NIHSS at 24 hours for a 1-point increase in the SES-WOA score. If a participant, for example, had the median NIHSS at 24 hours observed in this population (NIHSS 6), a participant with a 1-point higher SES-WOA score (which ranges from −1 to 1) would have a NIHSS that was 1.3 points lower (4.7, 95% CI 3.6–4.8). A similar nonsignificant trend was seen between the nSES and 2 radiologic outcomes, the FIV and reperfusion status. We argue that these are neither statistically significant, nor clinically significant results, indicating that patients with lower nSES were not necessarily in a less favorable neurologic condition after their initial treatment.

After examining the association between nSES and functional outcome through dividing the participants into nSES quartiles, we found that the occurrence of 2 patient characteristics (a history of diabetes mellitus and BMI) differed significantly. Diabetes mellitus was more common among those with the lowest nSES, and this group also had the highest median BMI. This is in line with results of previously published cohort-based and population-based studies.^17,18^ We chose not to adjust for comorbidities in our main analysis due to the potential mediation of these variables on the association between the nSES and the functional outcome at 90 days.^19^ However, we did adjust for individual comorbidities in a sensitivity analysis in eTable 8 in eAppendix 4, and found that it did not affect the conclusion drawn from the main analysis. Nevertheless, it should be considered that we could not account for frailty or all comorbidities (such as depression, dementia, and pulmonary disease), that could influence patient recovery due to data availability. Future research could also be enhanced by further information about the patients' home-environment, such as housing quality and support system composition, which could also potentially affect recovery.

A challenge for studies researching socioeconomic inequalities in clinical health outcomes is the comparison of study findings. This is partly due to the great variation in definitions of (and proxies for) socioeconomic status.^8^ For example, several studies have used factors that are health care system–specific, such as health-insurance ownership. Although the use of other single-indicator proxies for socioeconomic status, such as household income, is perhaps more widely applicable, they mainly take one dimension of a patient's socioeconomic status into account. Using a composite score allows us to consider multiple factors when determining nSES. This has the additional advantage of aiding us in the identification of neighborhoods that might benefit from interventions.

However, the potential limitations of this method are also important to consider. A neighborhood measure of SES is a generalized proxy and, therefore, could be vulnerable to the ecological fallacy because it could be influenced by the overall spread of socioeconomic factors between households living within neighborhoods. Combining multiple socioeconomic factors may lead to loss of information and could influence the interpretability of the results. When interpreting the results of this study, it should be considered that the SES-WOA score has a range of −1 to 1. The reported effect estimates represent the association between a 1-point change on the scale and the outcome measure. A 1-point change in the SES-WOA score represents a considerable difference in overall neighborhood affluence.

To assess the potential effect of clustering within hospitals, we preformed sensitivity analyses.^20^ However, we chose to not include this adjustment in our main analyses to reduce model complexity because clustering did not significantly affect the outcomes or alter the conclusions. The analyses did not account for clustering within postcodes as the vast majority of the 617 postcodes represented only included 1 patient and therefore did not have intracluster variability.

The proportional odds assumption was violated in the ordinal regression model examining the association between the nSES and functional outcome at 90 days. However, the cOR reported can still be viewed as an average association, and we believe that the results of this study signal a persisting health care inequality in stroke care that needs to be researched further. The shift in the mRS is consistently in the same direction for all dichotomizations of the mRS.^21^ The conclusion drawn from the results of the ordinal regression analysis is further supported by the outcome of the binomial regression analysis, which showed that there was a significant association between the nSES and regaining functional independence.

Previous research has mainly focused on the effect of comorbidities and (pre-)hospital care as potentially intervenable factors for the influence of nSES on stroke outcomes.^7,8^ However, since there was no association between the degree of neurologic deficit 24-hour postprocedure or the radiologic parameters examined and nSES, we argue that further research into potential inequalities in post-hospital stroke care is needed. The addition of patients' discharge destination and extent of poststroke (rehabilitation) care could have provided useful information for our analyses but were not recorded in our data sets. Since post-hospital stroke care is not centralized and also less protocolized, the care received could be highly variable between centers.

In this study, we focused on the nSES, meaning that the results cannot be directly translated to individual patient-level socioeconomic status. However, people living in more affluent neighborhoods tend to have greater health literacy,^22^ which could enable them to adhere more closely to and engage more effectively in the development of their treatment plans. Awareness of the availability of additional (rehabilitation) care, such as extraphysical therapy or in-home nursing care, as well as having the financial means to access these resources, might also be more common among those living in more affluent neighborhoods. Examining inequalities in access to and the quality of rehabilitation care and health education for patients with stroke living in more socioeconomically deprived areas could allow us to identify potentially intervenable factors.

From both the patients' and the societal standpoint, the importance of patients with stroke regaining their functional independence cannot be overstated. The aging of the population in high-income countries is expected to lead to a higher consumption of health care resources and increasing health care costs. This will inflate the care-load of the working age population and the capacity of communities to adapt to potential changes in health care availability.^23^ Health care expenditure is higher in patients with a greater degree of disability after treatment than patients who regain functional independence,^24^ highlighting the necessity of research into the factors contributing to the postponement of disability and frailty until later in life.

In conclusion, we found that although living in a more socioeconomically affluent neighborhood is associated with more favorable functional outcome at 90 days, whereas it is not significantly associated with reperfusion status, FIV, or NIHSS score at 24 hours. This provides evidence that nSES-based inequalities in regaining functional independence exist in a high-income country with centralized stroke care and adherence to a national stroke protocol. Focusing on inequalities in poststroke care is essential to move toward achieving health equity for patients with ischemic stroke.

## Appendix Coinvestigators

**Table:**

Coinvestigators are listed in the supplementary material at Neurology.org/N.

## Glossary

aOR: adjusted OR
BMI: body mass index
cOR: common OR
eTICI: expanded treatment in cerebral infarction
EVT: endovascular thrombectomy
FIV: follow-up infarct volume
GEE: generalized estimating equations
IQR: interquartile range
mRS: modified Rankin Scale
NIHSS: NIH Stroke Scale
nSES: neighborhood socioeconomic status
OR: odds ratio
RCT: randomized clinical trial
